# Differentiation Between Ulcerative Colitis and Crohn's Disease Using Abdominal Computed Tomography in Patients With First-Time Inflammatory Bowel Disease

**DOI:** 10.7759/cureus.59691

**Published:** 2024-05-05

**Authors:** Shinji Yamamoto, Nobukiyo Yoshida, Noriko Sakurai, Atsushi Ichikawa, Koji Takeshita, Yukinori Okada

**Affiliations:** 1 Department of Radiological Technology, Japan Community Healthcare Organization (JCHO) Tokyo Yamate Medical Center, Tokyo, JPN; 2 Department of Radiological Technology, Niigata University of Health and Welfare, Niigata, JPN; 3 Department of Radiological Technology, Nihon University Itabashi Hospital, Tokyo, JPN; 4 Department of Radiology, Japan Community Healthcare Organization (JCHO) Tokyo Yamate Medical Center, Tokyo, JPN; 5 Department of Radiology, St. Marianna University School of Medicine, Kawasaki, JPN

**Keywords:** computed tomography, subcutaneous fat area, visceral fat area, ulcerative colitis, crohn’s disease

## Abstract

Background

Ulcerative colitis (UC) and Crohn's disease (CD) are classified as inflammatory bowel diseases (IBDs). However, they have different pathogeneses and treatment strategies and need to be differentiated.

Purpose

To determine the feasibility of differentiating UC from CD in patients with first-time IBD based on simple abdominal computed tomography (CT) findings.

Methods

We conducted a retrospective study of patients diagnosed with IBD for the first time at our hospital between January and December 2021. Age, sex, white blood cell count, albumin concentration, C-reactive protein concentration, visceral fat area, subcutaneous fat area, and psoas major volume were extracted and used to differentiate the two groups.

Results

Forty-three patients were selected. Their mean age was 35.60 ± 17.19 years, and 32 were male, while 11 were female. The visceral fat cross-sectional area was 51.80 cm^2^ for UC and 21.10 cm^2^ for CD (p < 0.01). The subcutaneous fat cross-sectional area was 108.30 cm^2^ for UC and 66.30 cm^2^ for CD (p = 0.049). The total protein concentration was 6.15 g/L for UC and 6.60 g/L for CD (p = 0.012). Receiver operating characteristic curve analysis of the visceral and subcutaneous fat cross-sectional areas showed areas under the curve, 95% confidence intervals, sensitivities, and specificities of 0.750 and 0.675, 0.603-0.897 and 0.507-0.844, 0.810 and 1.00, and 0.591 and 0.409, respectively, at cutoffs of 26.53 and 36.6 cm^2^.

Conclusions

The visceral and subcutaneous fat cross-sectional areas determined with simple abdominal CT can differentiate UC from CD in patients with first-time IBD.

## Introduction

Inflammatory bowel disease (IBD) is a disease concept that includes ulcerative colitis (UC) and Crohn's disease (CD) [1]. IBD is a chronic, non-infectious inflammation of the gastrointestinal tract with a long-term course that results in decreased quality of life [2]. In contrast, patients with IBD associated with extensive colitis are at risk of colorectal cancer [3]. Clinical manifestations of IBD include abdominal symptoms, such as diarrhea and abdominal pain, and systemic symptoms, such as fever, weight loss, and inflammatory response. This puts patients at risk of undernutrition due to an imbalance between nutrition and caloric loss due to inflammation [4,5]. In contrast, if IBD is suspected based on clinical symptoms, a definitive diagnosis is made based on blood tests, imaging, endoscopy, surgical findings, and histopathology. Abdominal computed tomography (CT) is a routine part of IBD examination. From CT images, it is possible to obtain body composition data, such as body fat cross-sectional area, subcutaneous fat cross-sectional area, and psoas major volume. Muscle density can also be determined from the CT values of the psoas major without the need for additional imaging [6-8].

The distinction between UC and CD is essential because of their different treatment strategies and pathophysiology; UC is a disease confined to the colon, whereas CD can involve the entire gastrointestinal tract, except the colon. However, the difference between UC and CD is not solely related to the presence of lesions. Factors released from adipocytes have been reported to be involved in inflammation in CD, although this was a basic experiment, and signals released from mesenteric fat are involved in the worsening of inflammation [9]. Furthermore, an association between the mesenteric fat measured using CT and the severity of intestinal fiber stenosis has been reported in CD, and it is possible to predict the degree of fibrosis at the time of surgery [10]. These reports suggest that fat status may be useful in differentiating UC from CD.

This study aimed to determine whether body composition data obtained from CT images of patients with IBD, especially fat and muscle status, can be used to differentiate UC from CD.

## Materials and methods

Ethical considerations

This study was conducted after approval from the Ethics Committee of our hospital (permission number: J-176, July 4, 2023). As this was a retrospective case-observation study, it was difficult to obtain prior informed consent from the patients, and an opt-out for this study was enforced on our hospital website and at the Radiology Department reception desk.

Study design

This retrospective case-control study was conducted at Tokyo Yamate Medical Center, Japan.

Case selection

We included patients diagnosed with first-episode IBD at our clinic between January and December 2021 and during referral care periods who underwent simple CT of the abdomen, including the umbilical region, within three months before or after diagnosis.

The exclusion criteria were duplicate CT scans of the same patient, patients who had been diagnosed with IBD and were undergoing treatment or follow-up, patients who had received prior medication more than three months before the definitive diagnosis, and patients for whom more than three months elapsed between the diagnosis of IBD and CT.

The diagnosis of IBD was based on clinical data obtained from examination, history, blood tests, endoscopy, radiology, surgical findings, and pathological evaluation. UC and CD were diagnosed by a gastroenterologist based on diagnostic criteria [11].

CT protocol

All CT examinations were performed using two CT machines (Aquilion Prime 80 and Aquilion CX 64; Canon Medical Systems, Tochigi, Japan). The imaging parameters are listed in Table 1.

**Table 1:** Acquisition and reconstruction parameters for simple abdominal computed tomography for the diagnosis of inflammatory bowel disease. AEC: automatic exposure control, SD: standard deviation, FC: filter convolution. AIDR 3D eMild: adaptive iterative dose reduction three-dimensional enhance mild

	Protocol
Scan type	Helical
Tube potential	120 keV
Image quality parameter	SD 12
AEC	Volume EC
Rotation time (s)	0.5
Pitch	1.375:1
Z-axis coverage (rows × mm)	Aquilion PRIME SP 80×0.5
	Aquilion CX 64×0.5
Image slice thickness (mm)	2
Interval (mm)	0.5
Kernels	FC04
Reconstruction	AIDR 3D eMild
Length of scan	from the top of the diaphragm to the pelvis

Image analysis

All CT images were analyzed for body composition data by a single radiologist with more than 20 years of experience. The visceral fat area (VFA) and subcutaneous fat area (SFA) were automatically extracted from the abdominal CT images using an image processing system (Ziostation2, Amin Corporation, Tokyo, Japan). The total fat area (TFA) was calculated by adding VFA and SFA. Based on several previous studies, the radio density range for extraction was set from -160 to -70 HU, which is the default for the ZIO station [6,12]. In addition, the VFA/SFA and VFA/TFA ratios were calculated.

To evaluate the psoas major, its volume (cm^3^) and CT value (Hounsfield value) were calculated after automatically extracting its left and right parts from the same abdominal CT axial image using an imaging system. To compensate for the differences in the body sizes of the patients, the skeletal muscle mass index (SMI) was calculated by dividing the volume of the psoas major section by the square of the height (cm).

**Figure 1:**
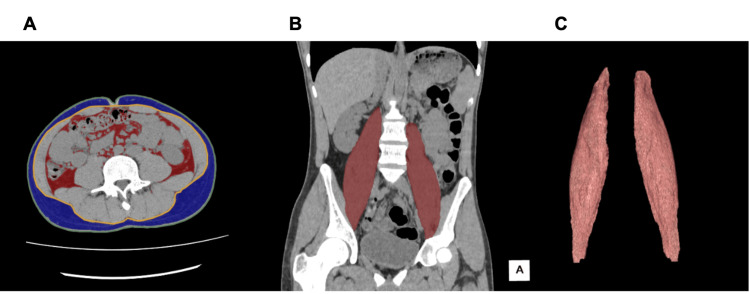
Segmentation and extraction of subcutaneous fat, visceral fat, and psoas major from an axial image obtained with simple abdominal computed tomography. (A) Extraction of visceral and subcutaneous fat. (B) Extraction of the psoas major. (C) Volume rendering image of the psoas major.

Figure 1 shows the extracted subcutaneous fat, visceral fat, and psoas major on an axial image from a simple abdominal CT scan.

Clinical findings

All clinical data, including age, sex, height, weight, BMI at the time of IBD diagnosis, and hematological data (total protein (g/L), albumin (g/L), and CRP (mL/L)), were obtained from the electronic medical records.

Statistical analysis

All statistical analyses were performed using Easy-R developed at the Omiya Medical Center of Jichi Medical University Hospital [13]. The Mann-Whitney U test was used to determine the association between radiological characteristics (visceral fat area, SFA, and psoas major volume) and clinical findings. Receiver operating characteristic (ROC) analysis was used to calculate the cutoff values and the area under the curve. Fisher’s exact test was used to confirm the distribution bias. Spearman's correlation coefficient was used to determine the relationships between the indicators. For all statistical tests, p < 0.05 was set as the level of statistical significance.

## Results

Baseline characteristics

From January to December 2021, 872 patients underwent abdominopelvic CT for the diagnosis of IBD. Of them, 828 were excluded based on the exclusion criteria; four had duplicate CT scans; 815 had been diagnosed with IBD and were undergoing treatment or follow-up; one had received prior medication more than three months before the definitive diagnosis; four underwent CT after more than three months after the diagnosis of IBD; three did not know they had IBD; and one patient had a complicated course.

Ultimately, 43 patients with first-episode IBD were selected, of whom, 21 had UC and 22 had CD. Their mean age was 35.60 years, and 32 were male while 11 were female. The demographic data of these patients are presented in Table 2.

**Table 2:** Baseline demographic characteristics of the cohort. SD: standard deviation, BMI: body mass index, VFA: visceral fat area, SFA: subcutaneous fat area, PMV: Psoas major volume, SMI: skeletal muscle mass index, CT: computed tomography, HU: Hounsfield unit, TP: total protein, ALB: albumin, CRP: C-reactive protein

Characteristic		n	Mean	SD
Age			35.6	17.2
Sex	Male	32		
	Female	11		
Height (cm)			168.5	8.7
Weight (kg)			59.8	11.1
BMI (kg/m^2^)			20.9	2.8
VFA (cm^2^)			44.6	37.6
SFA (cm^2^)			96.3	63.6
VFA/SFA			0.6	0.8
PMV (cm^3^)			399.7	150.5
SMI			6.6	2.4
CT value (HU)			48.0	8.1
TP (g/L)			6.3	0.8
ALB (mL/L)			2.9	0.6
CRP (g/L)			4.5	4.1

Clinical outcomes

The visceral fat cross-sectional areas were 51.80 and 21.10 cm^2^ for the UC and CD groups, respectively (p = 0.005). The subcutaneous fat cross-sectional areas were 108.30 and 66.30 cm^2^ for the UC and CD groups, respectively (p = 0.049). The total protein concentrations were 6.15 and 6.60 g/L for the UC and CD groups (p = 0.012). A comparison of the clinical data of patients with first-episode IBD is presented in Table 3.

**Table 3:** Comparison of the clinical data of patients with ulcerative colitis and Crohn’s disease. Statistical analysis: Mann–Whitney U test for significance and Fisher’s exact test for distributional bias. Spearman's correlation coefficients were used to determine correlations. BMI: body mass index, VFA: visceral fat area, SFA: subcutaneous fat area, PMV: psoas major volume, SMI: skeletal muscle mass index, CT: computed tomography, HU: Hounsfield unit, TP: total protein, ALB: albumin, CRP: C-reactive protein

		UC	CD	p-value
Number		21	22	
Age		41.0	28.5	0.107
Sex	Male	17	15	0.489
	Female	4	7	
Height (cm)		171.0	167.0	0.253
Weight (kg)		63.0	58.5	0.243
BMI (kg/m^2^)		22.1	21.1	0.409
VFA (cm^2^)		51.8	19.1	0.005
SFA (cm^2^)		108.3	66.3	0.049
VFA/SFA		0.5	0.3	0.294
PMV (cm^3^)		418.3	369.2	0.493
SMI		1.5	1.4	0.656
CT value (HU)		47.4	49.0	0.743
TP (g/L)		6.2	6.6	0.012
ALB (mL/L)		2.8	3.0	0.336
CRP (g/L)		3.8	4.6	0.627

**Figure 2:**
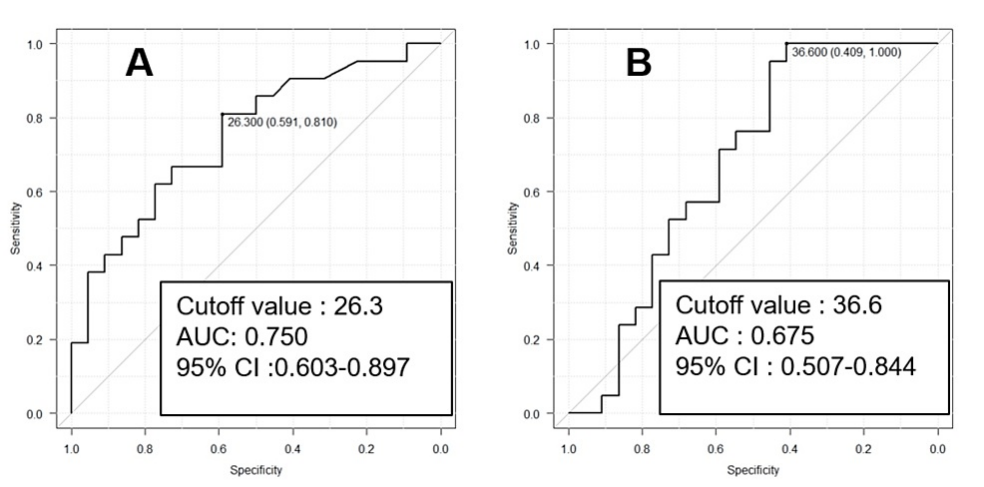
Results of receiver operating curve analysis of the visceral A and subcutaneous B fat areas in patients with first-time inflammatory bowel disease.

ROC analysis of the visceral fat cross-sectional area of the patients with primary UC and CD showed AUC, 95% confidence interval, sensitivity, and specificity of 0.750, 0.603−0.897, 0.810, and 0.591, respectively, at a cutoff of 26.30 cm^2^. In contrast, the AUC, 95% confidence interval, sensitivity, and specificity for the subcutaneous fat cross-sectional area were 0.675, 0.507−0.844, 1.000, and 0.409, respectively, at a cutoff of 36.60 cm^2^. The results of the ROC analysis of the visceral and subcutaneous fat areas in patients with first-time IBD are shown in Figure 2.

## Discussion

The results showed significantly lower visceral (p = 0.005) and subcutaneous (p = 0.049) fat in patients with CD than in those with UC. However, no statistically significant difference was observed in the psoas major volume (p = 0.493).

For cases of malignancy, cachexia and decreased muscle mass are observed. The L3 muscle mass, for example, is a good indicator [14]. Sarcopenia loss has also been reported in patients with heart failure and malignancy [15]. However, in the present study, the muscle mass did not differ between the UC and CD groups, indicating that fat may be involved in the pathogenesis of IBD.

Studies have reported the influence of adipose tissue on the pathogenesis of CD. Patients with CD accumulate intra-abdominal fat regardless of BMI, and this may be accompanied by mesenteric fat gain, which is characteristic of the development of creeping fat [16]. The VFA/SFA ratio was used to diagnose VF-type obesity. VF is metabolically active and is strongly associated with elevated serum concentrations of several proinflammatory cytokines such as IL-6 and tumor necrosis factor-α [17]. Creeping fat is also a hallmark of CD, and adipose tissue secretes growth factors such as adipocytokines, chemokines, and vascular endothelial growth factors. Several studies have reported that a high VFA/SFA ratio is a marker of progressive CD [17].

However, in the present study, visceral and subcutaneous fat loss was characteristic of CD in patients with first-time CD. Although the relationship between these two is difficult to determine, starvation may be involved in CD in addition to inflammation [9]. Changes in fat metabolism to compensate for the starvation and the increase in fat in the mesentery, as described above, may exacerbate inflammation; however, fat is consumed in CD, and it may also adhere to the mesentery and increase as a compensatory function.

In this study, CD was differentiated from UC based on the decrease in abdominal fat mass, suggesting the occurrence of fat abnormalities rather than cachexia and inflammation.

This study has a few limitations. First, the sample was very small due to the inclusion of only patients with first-time IBD to exclude confounding factors, and women with IBD were under-represented in the study. These factors were associated with errors in the analysis of the correlation between body composition data and IBD. Second, because this was a retrospective observational study, we could not completely rule out patient selection bias. Therefore, a larger study should be conducted to confirm our findings. Third, VFA evaluation also requires fat analysis of the entire abdomen, including the area around the inflammatory lesion in the intestine. Therefore, a prospective clinical trial with a larger sample is warranted.

## Conclusions

In conclusion, the composition data from abdominal CT scans revealed significant differences in visceral and subcutaneous fat cross-sectional areas as early indicators for the differentiation between CD and UC in patients with IBD. Combined with clinical findings and IBD diagnostic indices, these results suggest that a more accurate diagnosis can be made, and early treatment can be initiated. CD is a fatal disease resulting in a high degree of starvation. Fat management appears to be important when treating CD because fat gain worsens the condition.
